# Myostatin A55T Genotype is Associated with Strength Recovery Following Exercise-Induced Muscle Damage

**DOI:** 10.3390/ijerph17134900

**Published:** 2020-07-07

**Authors:** Jooyoung Kim, Kwanghoon Park, Joohyung Lee

**Affiliations:** 1 Office of Academic Affairs, Konkuk University, Chungju-si 27478, Korea; hirase1125@hanmail.net; 2 Department of Sport, Health and Rehabilitation, College of Physical Education, Kookmin University, Seoul 02707, Korea; niceradio@naver.com

**Keywords:** eccentric exercise, genotype, muscle damage, myostatin, strength recovery

## Abstract

Myostatin A55T genotype is one of the candidates showing inter-individual variation in skeletal muscle phenotypes. The aim of this study was to investigate the effect of the myostatin A55T genotype on markers of muscle damage after eccentric exercise. Forty-eight young, healthy male college students (age = 24.8 ± 2.2 years, height = 176.7 ± 5.3 cm, weight = 73.7 ± 8.3 kg) were enrolled in this study, and muscle damage was induced through 50 reps of maximal eccentric muscle contraction. As markers of muscle damage, maximal isometric strength (MIS), muscle soreness, creatine kinase (CK), and aspartate transaminase (AST) were measured. Myostatin A55T genotypes were classified into homozygous myostatin A55T allele (AA, n = 34, 72%), heterozygous myostatin A55T allele (AT, n = 13, 26%), and homozygous mutant carriers (TT, n = 1, 2%). After eccentric exercise, the subjects with heterozygous for AT showed markedly quicker MIS recovery compared to the AA group (*p* = 0.042). However, there were no significant variations in muscle soreness (*p* = 0.379), CK (*p* = 0.955), and AST (*p* = 0.706) among the groups. These results suggest that AT in myostatin A55T genotype may be associated with quicker strength recovery following exercise-induced muscle damage.

## 1. Introduction

Repetitive, high-intensity, eccentric muscle contractions cause muscle damage [1]. It produces muscle disruption and excitation–contraction coupling gets impaired, and inflammation and muscle protein degradation occurs [2,3]. Therefore, maximal isometric strength (MIS) is reduced after muscle damage, and the protein is increased, such as creatine kinase (CK) and aspartate transaminase (AST), in the blood with delayed onset muscle soreness (DOMS) [4,5]. 

According to a number of studies, there is an inter-individual variation in the response of muscle damage that appears after eccentric exercise, which has been reported to be affected by age, sex, body composition, training status, and genetic polymorphism, etc. [6,7,8,9,10]. Especially, it was presented as an evidence that several genetic polymorphisms can play an important role in the response to muscle damage [11,12,13]. 

Myostatin, which is also known as growth/differentiation factor-8 (GDF-8), is one of the tumor growth factor (TGF-β) superfamily [14]. Myostatin is known to combine with activin type IIB (ACVR2B) and type IB (ACVRB) receptors and activate Smad2/3, p38 MAPK, and Erk1/2 signal transduction pathways [15] to control muscle growth and repair by inhibiting proliferation and differentiation of the satellite cell, which is also called muscle stem cell [16]. Myostatin has been extensively studied in human and animal models [17,18], and single nucleotide polymorphism (SNP) has also been investigated in relation to sarcopenia or obesity [19,20]. A55T (rs180565, 163 G > A) is a representative myostatin SNP identified from human genomic studies [20]. Myostatin A55T polymorphism is located within the 42-115 amino acid sequence in the inhibitory domain of the myostatin precursor, and it plays an important role for the stability of GDF-8 propeptide inhibitory activity and it influences mature myostatin [21].

In a study in the exercise science field, it was reported that AT and TT among the myostatin A55T showed a larger muscle hypertrophy than AA after 8 weeks of resistance exercise [22]. This result suggests that the specific type among the myostatin SNP is a potential candidate that can positively affect the skeletal muscle phenotype after resistance exercise. However, previous myostatin SNP studies mainly observed chronic effects such as muscle hypertrophy after resistance exercise [22,23], and there was no study about acute response such as muscle damage. 

To our knowledge, this study is the first to investigate myostatin A55T genotype and exercise-induced muscle damage targeting the Korean population. Therefore, the purpose of this study was to investigate the potential effects of the myostatin A55T genotype on muscle damage indices such as MIS, muscle soreness, CK, and AST after eccentric exercise.

## 2. Materials and Methods

### 2.1. Subjects

Forty-eight healthy male college students (age = 24.8 ± 2.2 years; height = 176.7 ± 5.3 cm; weight = 73.7 ± 8.3 kg) participated in this study. The sample size of this study was calculated based on previous studies [21,24]. All subjects did not have regular strength training in the last six months, were non-smokers, and had not taken any anti-inflammatory drugs, vitamins, or protein supplements recently. They also did not have current musculoskeletal or nerve related disease. In addition, baseline CK of all subjects were all within the normal range (male: 24–195 UL). All subjects were fully informed by the investigator regarding the purpose and procedure of the study and agreed to limit participation in excessive exercise or physical activity as well as analgesic intake or alcohol consumption during the study. All subjects gave their informed consent for inclusion before they participated in the study. The study was conducted in accordance with the Declaration of Helsinki, and the protocol was approved by the Ethics Committee of Kookmin University (number: KMU-201312-HRBR-005-02).

### 2.2. Eccentric Exercise

The elbow flexor model was used for the eccentric exercise in this study. This model has been used in many exercise-induced muscle damage studies [25,26,27]. The subjects performed eccentric exercise on a modified preacher curl machine (EMC model, Kookmin University, Seoul, Korea). The subjects were first seated on a bench of a modified preacher curl machine and then they placed a non-dominant arm on the pad. Then, when the investigator lowered the lever attached to the pad, the subjects were made to resist this movement and tried to maximally contract the elbow flexor muscles [28]. One muscle contraction lasted for 3 s and then rested for 12 s. There were 25 muscle contractions per set and 2 sets were performed in total (total 50 times performed). The rest period between sets was 5 min. All protocols related to the eccentric exercise in this study were based on previous studies [1,25,26].

### 2.3. Myostatin A55T Genotyping 

DNA was extracted according to the manufacturer protocols by using Nucleo-Spin Blood kit (NucleoSpin Blood kits, Macherey-Nagel, Inc., Germany). After injecting 25 μL proteinase K and 200 μL buffer into the blood samples, it was stored in the warm bath (70 °C) for 10–15 min. Afterwards, 210 ll ethanol (96–100%) was added and then mixed again. Then, 100 μL buffer was injected after two wash processes, and the samples were kept at room temperature for one minute, and centrifuge for one minute. Myostatin A55T genotype used real-time polymerase chain reaction (Real-Time PCR, iQ-5 Multi-Color Real-time PCR Detection System, BIO-RAD, USA). The real-time PCR condition in this study is shown as follows. First, after 5 min of pre-denaturation at 94 °C, the DNA denaturation was performed by cycling 34 times at 94 °C for 60 s. Then, primer annealing was performed for 120 s at 56 °C, and 35 cycles of DNA polymerization and extension for 90 s at 72 °C, and last extension for 8 min at 72 °C were performed. The primer sequences in this study were prepared according to the study of Kostek et al. [23], which are 5′-TGCAATGTACTTGGAGAGACAAAACAT-3′ (forward primer), 5′-GCTGTTTCCAGAGGAAGTTTACTGATC-3′ (reverse primer).

### 2.4. Maximal Isometric Strength 

Strength loss after stretching is measured by isometric force at single muscle length or single joint angle [29]. The MIS measurement method of this study referred to the previous studies [23,26,30]. The MIS of elbow flexor muscles was measured by using a strain gauge (Jackson Strength Evaluation System Model. 32628CTL, Lafayette Instrument Company, Lafayette, IN, USA) attached to the modified preacher curl machine [23]. The investigator calibrated the strain gauge before each test and placed the subject’s arm to be tested at 90 degrees. The other arm of the subject was placed on the knee. At the investigator’s signal, the subjects contracted the arm to their maximum force. Each contraction lasted for 3 s and one minute of rest was allowed between contractions. MIS was measured three times at each time period (before, immediately after, 24, 48, 72, and 96 h after exercise), and one-minute rest was given between measurements. After measuring three times, the average value was calculated and used.

### 2.5. Muscle Soreness

Visual analogue scale (VAS) was used to measure muscle soreness. The high reliability of VAS has been known [31] and it is known as a suitable tool for quantitative assessment of acute muscle soreness and observation of clinical changes of muscle soreness [32]. The VAS has a constant line of 100 mm, the left first position (0 mm) indicates painless state and the right end (100 mm) indicates severe pain. The VAS was distributed to the subjects, and they vertically marked the degree of pain felt at each measurement period [30]. 

### 2.6. Creatine Kinase and Aspartate Transaminase 

First, 5 mL of blood samples were collected each time from antecubital vein using a BD serum collection vacutainer. The subjects kept fasting for 8 h prior to blood collection and were recommended to have enough fluid intake. Blood was placed in tubes and centrifuged 2500–3000 rpm for 10 min at 4 °C. Samples were stored at −80 °C until analysis, and CK and AST were analyzed according to manufacturer protocols using an automatic blood analyzer (VITROS DT60II, Ortho Clinical Diagnostics, NJ, USA). 

### 2.7. Statistical Analysis

All data in this study were described using means and standard deviations (SD). To verify the normal distribution of the myostatin A55T genotype, a Hardy–Weinberg equilibrium test was performed using X^2^. Repeated measure one-way analysis of covariance (ANOVA) was performed to analyze the difference of the muscle damage index in each time period after eccentric exercise according to the Myostatin A55T genotype (AA vs. AT), and post hoc test used Bonferroni. The SPSS program (version 18.0, IBM, Armonk, NY, USA) was used for all statistical analysis. The statistical significance level was α < 0.05.

## 3. Results

### 3.1. Hardy–Weinberg Equilibrium Test of Myostatin A55T Genotype

In this study, the myostatin A55T genotype was classified as homozygous myostatin A55T allele (AA, n = 34, 72%), heterozygous myostatin A55T allele (AT, n = 13, 26%), and homozygous mutant carriers (TT, n = 1, 2%), respectively. This study only compared and analyzed AA and AT among the myostatin A55T genotypes, and TT was excluded (Table 1). The Hardy–Weinberg equilibrium test result in this study verified the gene distribution equilibrium of the myostatin A55T genotype (X^2^ = 0.02, *p* = 0.849).

### 3.2. Change of Muscle Damage Markers After Eccentric Exercise According to Myostatin A55T Genotype

Changes in maximal isometric strength after eccentric exercise showed a significant interaction between time and group between AA and AT of the myostatin A55T genotype (*p* = 0.042). The maximal isometric strength in AA and AT decreased and then recovered immediately after eccentric exercise, but there was a 23.3% difference in muscle recovery between the two genotypes 72 h after exercise, and AT recovered faster than AA (*p* = 0.041, Table 2). 

However, changes in muscle soreness after eccentric exercise was no significant interaction in time and group between AA and AT (*p* = 0.379, Table 3). In addition, changes in CK (*p* = 0.955, Table 4) and AST (*p* = 0.706, Table 5) was no significant interaction in time and group between AA and AT of the myostatin A55T genotype after eccentric exercise.

## 4. Discussion

We tested how the A55T genotype, which is one of the myostatin SNPs, affects the index of muscle damage such as MIS, muscle soreness, CK, and AST after eccentric exercise. After the eccentric exercise, AT showed faster MIS recovery than AA among the myostatin A55T genotypes, which is a very interesting result. 

Eccentric exercise-induced muscle damage causes initial damage (Z-disc streaming) and secondary damage (inflammatory response, neutrophil infiltration, and macrophage phagocytosis), and muscle remodeling progresses afterward [11]. Muscle remodeling occurs by activation, proliferation, and differentiation of a quiescent satellite cell located around the muscle fiber after muscle damage [33]. The satellite cell proliferates after 24–48 h and then moves to the site where the damage occurred and supports the repair process to help strength recovery [34,35]. Especially, myostatin may significantly influence the function of the satellite cell, and myostatin reduces satellite cell activation and differentiation by suppressing muscle regulatory factors such as Pax7 and MyoD [36]. 

Several studies have reported that muscle hypertrophy may be significantly different according to myostatin genetic polymorphism after resistance training [22,23], and Li et al. [22] explained the potential cause of these findings as because the location of myostatin genetic polymorphism interferes with the ability of protein products to inhibit muscle growth in untrained conditions. Myostatin A55T polymorphism is related to the regulatory properties of GDF-8 propeptide [37,38], and GDF-8 propeptide has a function to inhibit GDF-8 biological activity [39]. A few animal studies showed that the overexpression of GDF-8 propeptide enhances muscle development [40,41]. This suggests that there may be a difference in GDF-8 propeptide activity among the myostatin A55T genotypes, and that AT will be higher in GDF-8 propeptide activity than AA among the myostatin A55T genotypes. This is thought to be a potential mechanism for different strength recovery of AT and AA, affecting satellite cell function and muscle remodeling after muscle damage. Li et al. [22] also found that the thickness of biceps was significantly increased after 8 weeks of training in individuals with AT in the myostatin A55T genotype. Unfortunately, to the best of our knowledge, there are no studies that have directly identified GDF-8 propeptide activity or myostatin levels according to the myostatin A55T genotype. Therefore, this hypothesis needs to be investigated in future studies. 

On the other hand, no significant differences were found in muscle soreness, CK, and AST according to myostatin A55T genotype after eccentric exercise, which contrasts with previous studies [25,26]. This discrepancy may have been influenced by factors such as sex. In the previous studies that showed significant differences in muscle soreness or blood markers, such as CK according to genotype after eccentric exercise, included females in the study subjects [25,26,28]. Sex is one of the potential factors that can influence the results of genetic polymorphism research. Several studies have reported that female estrogens may play a protective role in attenuating muscle damage caused by eccentric exercise because they have anti-oxidant characteristics and membrane stability properties [42,43]. In addition, differences in ethnic, sample size, training, body mass, and physical activity levels can be the potential causes of different results from previous studies [11,12,44].

There are some limitations in this study. First, the subjects of this study were general people, not well-trained athletes. Additionally, eccentric exercise was also performed with the elbow flexor only. In addition, small number of subject samples and low frequency homozygous mutant carriers (AA) may limit statistical power in the analysis. In future studies, larger sample sizes will need to be added. Finally, only a few muscle damage indices were measured after eccentric exercise. Therefore, there is a limitation in confirming the mechanism of relation between specific genotype among myostatin A55T genotype and strength recovery after eccentric exercise. Some of the parts described in the discussion are only hypotheses based on previous research. In future studies, various indicators related to myostatin may need to be measured. For example, GDF-8 propeptide activity or myostatin levels may be different depending on the human myostatin A55T genotype, which should be checked for actual differences and their relationship with muscle injury indices. 

## 5. Conclusions

In this study, AT of the myostatin A55T genotype was associated with rapid recovery of MIS after eccentric exercise. Therefore, the specific genotype of myostatin A55T may have a potential effect on the recovery of MIS observed after muscle damage by eccentric exercise. In the future, more Korean populations will need to be investigated for the relationship between myostatin A55T and strength recovery after muscle damage.

## Figures and Tables

**Table 1:** Subject characteristics according to the myostatin A55T genotype.

Variable	AA (n = 34, 72%)	AT (n = 13, 26%)
Age (years)	25.0 ± 2.2	24.3 ± 2.1
Height (cm)	177.1 ± 4.8	175.2 ± 6.8
Weight (kg)	74.5 ± 7.7	70.7 ± 9.8
Body fat (%)	16.8 ± 5.8	15.4 ± 4.0

Values are means ± SD; There were no significant differences between groups.

**Table 2:** Changes in maximal isometric strength (MIS) after eccentric exercise according to the myostatin A55T genotype.

Unit: %	Pre	Post	24 h	48 h	72 h	96 h	*p*
AA (n = 34)	100 ± 0.0	43.6 ± 3.8	55.4 ± 4.6	59.0 ± 5.2	62.8 ± 5.8	74.0 ± 6.1	0.042
AT (n = 13)	100 ± 0.0	55.4 ± 6.2	69.1 ± 7.5	76.1 ± 8.4	86.2 ± 9.4 *	93.1 ± 9.8	

Values are means ± SD. * Significant between group (*p* < 0.05).

**Table 3:** Changes in muscle soreness after eccentric exercise according to the myostatin A55T genotype.

Unit: mm	Pre	24 h	48 h	72 h	96 h	*p*
AA (n = 34)	0 ± 0.0	35.7 ± 2.9	43.7 ± 2.7	37.1 ± 2.9	23.0 ± 2.5	0.379
AT (n = 13)	0 ± 0.0	33.7 ± 4.8	41.7 ± 4.4	34.2 ± 4.7	26.4 ± 4.1	

Values are means ± SD.

**Table 4:** Changes in creatine kinase (CK) after eccentric exercise according to the myostatin A55T genotype.

Unit: U/L	Pre	24 h	48 h	72 h	96 h	*p*
AA (n = 34)	142.6 ± 19.0	1103.4 ± 579.6	3911.3 ± 1321.9	7854.5 ± 1785.6	9569.4 ± 1680.0	0.955
AT (n = 13)	119.8 ± 30.7	883.8 ± 937.4	3354.2 ± 2137.8	8158.7 ± 2887.7	10868.5 ± 2716.9	

Values are means ± SD.

**Table 5:** Changes in aspartate transaminase (AST) after eccentric exercise according to the myostatin A55T genotype.

Unit: U/L	Pre	24 h	48 h	72 h	96 h	*P*
AA (n = 34)	29.1 ± 4.1	38.7 ± 6.3	78.6 ± 15.4	135.0 ± 22.2	181.0 ± 23.6	0.706
AT (n = 13)	22.9 ± 6.6	32.6 ± 10.3	74.2 ± 24.9	162.4 ± 36.0	201.4 ± 28.2	

Values are means ± SD.
